# High-quality multimodal MRI with simultaneous EEG using conductive ink and polymer-thick film nets

**DOI:** 10.1088/1741-2552/ad8837

**Published:** 2024-11-05

**Authors:** Nicholas G Cicero, Nina E Fultz, Hongbae Jeong, Stephanie D Williams, Daniel Gomez, Beverly Setzer, Tracy Warbrick, Manfred Jaschke, Ravij Gupta, Michael Lev, Giorgio Bonmassar, Laura D Lewis

**Affiliations:** 1 Graduate Program in Neuroscience, Boston University, Boston, MA, United States of America; 2 Department of Biomedical Engineering, Boston University, Boston, MA, United States of America; 3 Department of Electrical Engineering and Computer Science, Massachusetts Institute of Technology, Cambridge, MA, United States of America; 4 Institute for Medical Engineering and Science, Massachusetts Institute of Technology, Cambridge, MA, United States of America; 5 Athinoula A. Martinos Center for Biomedical Imaging, Department of Radiology, Massachusetts General Hospital, Charlestown, MA, United States of America; 6 Department of Radiology, Harvard Medical School, Boston, MA, United States of America; 7 Neurobiology Research Unit, Copenhagen University Hospital, Rigshospitalet, Copenhagen, Denmark; 8 Brain Products GmbH, Gilching, Germany

**Keywords:** multimodal imaging, electroencephalogram, MRI, artifacts, image quality

## Abstract

**Objective.:**

Combining magnetic resonance imaging (MRI) and electroencephalography (EEG) provides a powerful tool for investigating brain function at varying spatial and temporal scales. Simultaneous acquisition of both modalities can provide unique information that a single modality alone cannot reveal. However, current simultaneous EEG-fMRI studies are limited to a small set of MRI sequences due to the image quality and safety limitations of commercially available MR-conditional EEG nets. We tested whether the Inknet2, a high-resistance polymer thick film based EEG net that uses conductive ink, could enable the acquisition of a variety of MR image modalities with minimal artifacts by reducing the radiofrequency-shielding caused by traditional MR-conditional nets.

**Approach.:**

We first performed simulations to model the effect of the EEG nets on the magnetic field and image quality. We then performed phantom scans to test image quality with a conventional copper EEG net, with the new Inknet2, and without any EEG net. Finally, we scanned five human subjects at 3 Tesla (3 T) and three human subjects at 7 Tesla (7 T) with and without the Inknet2 to assess structural and functional MRI image quality.

**Main results.:**

Across these simulations, phantom scans, and human studies, the Inknet2 induced fewer artifacts than the conventional net and produced image quality similar to scans with no net present.

**Significance.:**

Our results demonstrate that high-quality structural and functional multimodal imaging across a variety of MRI pulse sequences at both 3 T and 7 T is achievable with an EEG net made with conductive ink and polymer thick film technology.

## 1. Introduction

Multimodal neuroimaging using simultaneous electroencephalography (EEG) and magnetic resonance imaging (MRI) has become an increasingly powerful approach in neuroscience research. Combining MRI’s high spatial resolution with EEG’s high temporal resolution allows for the concurrent acquisition of data spanning spatiotemporal scales (Huang-Hellinger *et al* 1995, Goldman *et al* 2000, Ritter and Villringer 2006, Mulert and Lemiuex 2023). Multimodal human neuroimaging with simultaneous EEG-MRI provides the advantage of producing two complementary datasets with the same underlying neural activity (Scrivener 2021, Warbrick 2022). Furthermore, simultaneous imaging provides unique insight into spontaneous changes in brain activity, such as the dynamics that arise during sleep (Dang-Vi *et al* 2008, Dresler *et al* 2012, Marawar *et al* 2017, Fang *et al* 2019, Fultz *et al* 2019, Setzer *et al* 2022), as well as across many other areas of neuroscience research (Morillon *et al* 2010, Walz *et al* 2013). However, simultaneous EEG-MRI studies to date have primarily been limited to studying the blood oxygenation level-dependent (BOLD) signal with conventional fMRI sequences. Due to both safety considerations and image artifacts, commercial EEG caps are strictly limited to specific scan protocols. Simultaneous EEG-MRI has, therefore, not been reported successfully with a range of other sequences that can provide distinct scientific and clinical information.

The safety profile and image quality associated with current EEG nets during fMRI scanning have been well characterized (Angelone *et al* 2004, Graf *et al* 2007, Luo and Glover 2011, Foged *et al* 2017, Hawsawi *et al* 2017, Poulsen *et al* 2017, Atefi *et al* 2019, Warbrick 2022). Current commercially available MR-conditional EEG nets are only cleared for use with sequences that have low power (falling under strict B1 + rms or specific absorption rate (SAR) limits (Faulkner 2016, Warbrick 2022)) and, therefore, are typically only used with T1 imaging and gradient-echo echo planar imaging (EPI) fMRI sequences, limiting the types of questions that can be investigated (Poulsen *et al* 2017). Many other pulse sequences provide valuable information in research and clinical settings, such as diffusion imaging to quantify water movement in the brain, fluid-attenuated inversion recovery (FLAIR) sequences, which have been used to identify microstructural changes in brain tissue (Bahsoun *et al* 2022), and susceptibility-weighted imaging (SWI), which is sensitive to compounds that distort the local magnetic field, such as calcium and iron, and is a valuable tool for visualizing venous structure and iron in the brain (Halefoglu and Yousem 2018). Furthermore, the availability of ultrahigh field MRI at 7 Tesla (7 T) is rapidly expanding, and these scanners provide higher sensitivity and improved spatial resolution (Balchandani and Naidich 2015, Biagi *et al* 2017, Trattnig *et al* 2018, Vachha and Huang 2021). However, higher magnetic field MRI scanners introduce concerns for EEG, such as localized radiofrequency (RF)-induced heating and substantially worse MR imaging artifacts than at 3 T, and require the development of dedicated EEG cap technologies (Jorge *et al* 2015). As ultra-high field scanners become increasingly prevalent (Balchandani and Naidich 2015, Dumoulin *et al* 2018, Vachha and Huang 2021) and advances in gradient strength and pulse sequences continue to develop, an EEG net is needed that can provide good image quality across a broad range of MRI modalities.

Beyond neuroscientific research, an EEG net compatible with diverse MRI modalities would also be helpful in clinical practice. Patients undergoing EEG monitoring in the intensive care unit frequently need to undergo MRI scans for clinical evaluation. Continuous EEG monitoring is often essential to enable the detection and tracking of abnormal neural activity, such as for continuous monitoring of seizures in patients with severe epilepsy (Claassen *et al* 2004), and removing the EEG net for an MRI scan carries the risk of missing clinically significant EEG information. Additionally, multimodal neuroimaging using functional MRI (fMRI) and EEG has recently shown clinical utility in predicting outcomes of patients with acute disorders of consciousness (Owen *et al* 2006, Claassen *et al* 2019, Leandro *et al* 2021, Amiri *et al* 2023a, 2023b). However, conventional EEG nets must be removed prior to any MRI and computed tomography (CT) imaging (Balasubramanian *et al* 2017) because they can severely impact image quality. As MRI technologies are starting to become more portable and have the potential to be integrated directly into the bedside (Wald *et al* 2020, Cooley *et al* 2021, Mazurek *et al* 2021), the need for MRI-conditional EEG nets will become increasingly important in clinical settings (Lemieux *et al* 1997, Ney *et al* 2013, Merhar and Chau 2016).

The primary source of artifacts when imaging conventional EEG nets is their copper wires, which connect the EEG electrodes to the amplifier. EEG electrodes and leads can create induced currents, shielding, and other perturbations to the electromagnetic fields within the MRI environment that can significantly reduce image quality, causing hyperintensities near electrodes, signal dropout, and other major artifacts that cannot be corrected for following image acquisition (Bonmassar *et al* 2001, Vasios *et al* 2006, Mullinger *et al* 2008a, 2008b). Recent technological advances in EEG hardware have led to a new type of EEG net that replaces these copper wires with conductive ink printed on polymer thick film (PTF), termed the ‘Inknet’ (Poulsen *et al* 2017). Prior work explicitly tested the safety profile of conductive ink-based EEG nets and found that these nets enable safe imaging even with a high-SAR sequence in normal operating mode (Poulsen *et al* 2017). However, whether this technology allows for high-quality imaging across a range of MRI pulse sequences and 7 T MRI has not yet been tested in human subjects.

Using PTF technology, we constructed a new net, the Inknet2, with a modular design to improve practical use, and tested its effects on image quality across several MRI pulse sequences. We found that Inknet2 enables high-quality imaging with a broad range of MRI sequences at 3 T and 7 T. The Inknet2 represents the first EEG hardware evaluated in humans across a wide range of imaging sequences. This capability enables the use of diverse anatomical and fMRI sequences with simultaneous EEG, allowing new types of multimodal imaging studies to be performed.

## 2. Methods

Four studies were performed to assess the image quality of the Inknet2: (1) numerical simulations to assess RF-induced artifact and current density estimations from the Inknet2, (2) phantom CT and 3 T MRI scans to compare image quality between the Inknet2 and conventional copper Cu-Net, (3) human 3 T MRI scans to compare anatomical image quality with and without the Inknet2, and (4) human 7 T MRI scans to compare anatomical and functional image quality with and without the Inknet2. The Institutional Review Board at Massachusetts General Hospital approved all prospective human study procedures, and secondary use of clinical data (figure 3(a)).

### EEG nets.

**Figure 1.**
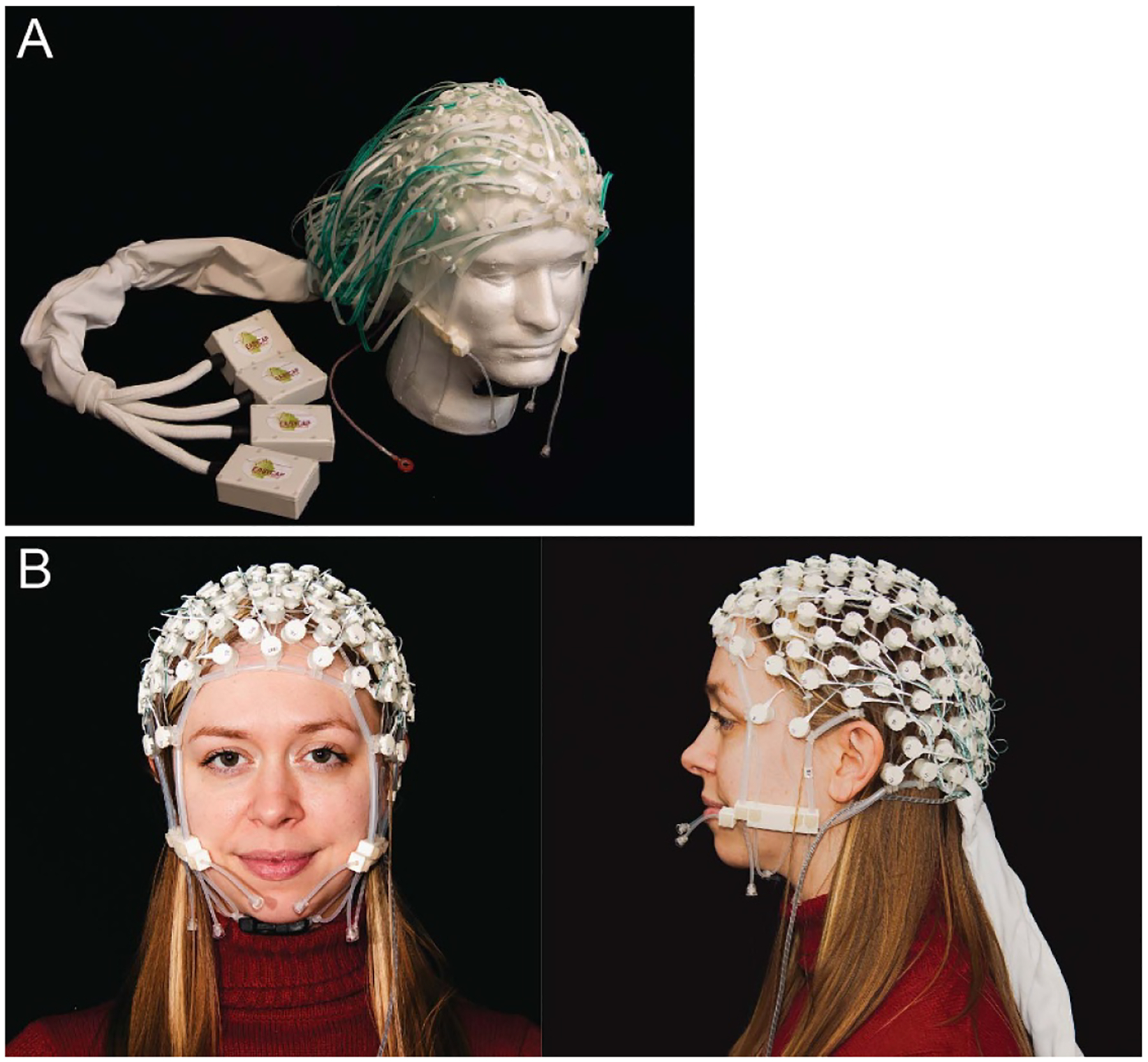
The Inknet2 128-channel EEG net.( A) The prototype Inknet2 128-channel EEG net. The experiments reported here were performed using this prototype. (B) For demonstration purposes, the latest prototype of the Inknet2 128-channel EEG net with minor modifications to cable routing to increase ease of application.

Phantom experiments compared two MR-conditional EEG nets: the Inknet2 and the conventional copper Cu-Net. The Inknet2 was a custom-made 128-channel MR-conditional EEG net (EasyCap GmbH, Herrsching, Germany; figure 1) that replaced the conventional copper leads with 128 conductive ink traces printed with PTF. The design was updated relative to the original Inknet (Poulsen *et al* 2017), in this case making all traces at the same length of 23 cm (mean trace resistance = 20.49 kΩ, SD = 0.85 kΩ). Relative to the original Inknet, the Inknet2 had improved cable routing, with updated trace lengths and geometry to allow easy application and connection of the electrodes, and aesthetic modifications such as improved bundling around cables and different chin adjustment straps. Conductive pads and leads ink (Engineered Conductive Materials, OH, USA) were screen-printed on 76.2 *μ*m thick *Melinex polyester PET* film (DuPont Teijin Films US Limited Partnership, VA, USA) and laminated to 152.4 *μ*m thick thermoplastic polyurethane film. The Inknet2 circuits fit in a commercial elastomer structure, and the PTF leads were passed near the subjects’ bodies and were connected to a 128-channel Brain Products amplifier system. The Cu-Net used for comparison was a commercially available 128-channel MR-conditional Geodesic Sensor Net, HCGSN 220 MR (MagStim). The leads were made of copper wires (mean lead resistance = 1 Ω) with 10 kΩ resistors used for the electrode connection. 3 T MRI RF safety tests of the Inknet2 were done with an adult-sized head phantom in order to ensure safety prior to use on human subjects (supplemental figure 2). To confirm typical EEG recording quality, Inknet2 EEG signals were measured outside the scanner while a human participant alternated between eyes open and closed states (supplemental figure 3). The EEG multi-taper spectrogram is shown after filtering between 0.1–30 Hz and re-referenced to average (tapers = [3 5], moving window = [5 1]) (supplemental figure 3(a)). Power from the multi-taper spectrogram was averaged across periods of eyes open and closed, respectively, to compute the average spectra for each condition (supplemental figure 3(b)).

### MRI RF safety tests.

The RF safety of the Inknet2 was tested in a 3 T MRI (Prisma, Siemens Healthineer) using an adult head-sized agar phantom. The dielectric properties of the phantom were selected to be similar to the adult brain properties at 128 MHz (Duan *et al* 2014, Hasgall *et al* 2018) (i.e. *σ* = 0.52 S m^−1^, *ε*_r_ = 65.4). Heating was measured using 8-channel fiber optic probes (OSENSA Innovations Corp., Coquitlam, BC, Canada), which were positioned at distributed locations across the Inknet2, including three hot spots estimated from the thermal simulation (Atefi *et al* 2019). The thermal paste was used to allow the fiber optic probes to be in contact with the surface of the agar phantom, and the EEG electrode was used to assess the RF-induced heating by scanning it with the EEG net. A high-power turbo spin-echo sequence (21 slices,0.9 × 0.9 × 5.0 mm voxels, TR/TE = 7600/86 ms, FA = 120°, average: 20) delivered 100% SAR for 30 min (SAR_head_: 2.85 W kg^−1^, 10gSAR_torso local_: 9.99 W kg^−1^) was used to produce the maximum allowed RF safety limit in a clinical scan. A 30-minute scan was performed, and the maximum temperature rise was 0.96 °C, which is the condition where no rationale is needed for the SAR label of a 1 h scan in normal operating mode as per the FDA guidance document (US Food and Drug Administration 2021) (supplemental figure 2).

### Numerical simulations.

Electromagnetic simulation results were conducted with Sim4Life (ZMT, Switzerland) with the Duke human model (v3.0) (Gosselin *et al* 2014) with EEG nets on the 3 T body transmit coil (diameter/length: 610/610 mm) with an RF shield. The 128-channel EEG traces were drawn around the head (trace width/length: 1/510 mm), with the criteria that each trace does not touch the others and exits along the right shoulder, with the dielectric properties of Inknet2 (*σ*: 25.55 S m^−1^, *ε*_r r_ = 4.2) and Cu-Net (*σ*: 5.8 × 10^7^ S m^−1^, *ε*_r_:1) (Jeong *et al* 2021). The numerical model of the EEG net was composed of electrodes (i.e. sponges), stems (not included in the model), and EEG traces. The trace in the Inknet2 was resistive (*R*: 20 kΩ) to minimize RF-induced heating and artifact during MRI. The properties of the sponges (i.e. electrodes) soaked in the KCl solution were *σ* = 2.14 S m^−1^, *ε*_r_ = 84.7, respectively (Atefi *et al* 2019), and positioned perpendicular to the model skin (diameter/length: 7/12 mm). The current limiting resistors (*R*: 10kΩ) were added between the electrode and trace in the case of the Cu-Net (Krakow *et al* 2000). A high-performance GPU (V100 32GB, NVIDIA, Medford, MA) was used to accurately sample the trace with the maximum grid step of 0.8 × 0.8 × 1.0 mm, and a minimum grid step of 0.4 × 0.5 × 0.4 mm was set to ensure the space and connection between trace and electrode, resulting in the total number of 516.73 MCells. The simulation results were normalized to fields that produced 2 *μ*T at the coil center in the case of No-Net, which is the approximated field strength to create a 90° flip angle in ^1^H with a rectangular RF pulse for the 3 ms duration (Collins *et al* 1998). The same normalization factors were applied to the Inknet2 and Cu-Net cases for comparison.

The current density maps (*J*_RMS_ [A m^−2^]) were displayed along with the two EEG trace scenarios with a 3D surface view to show the relative RF-induced current density on the trace and the subject’s head (supplemental figure 4). The fields were normalized to generate 3.2 W kg^−1^ in the head to demonstrate the worst-case scenario. In the case of a conductive EEG net with current limiting resistors, the figure shows that it could mitigate the current flows that go into the EEG electrodes but not entirely block the induced current, which results in current flows in the tissue (supplemental figure 4(d)). The current density on the Inknet2 was relatively small, and thus the delivered current on the tissue was not noticeable (supplemental figure 4(c)).

### Image acquisition.

To assess image quality with the Inknet2 across a range of pulse sequences, we tested multiple sequences in a phantom and in humans with and without the EEG nets. 3 T scans were acquired using a Siemens Magnetom Skyra with a 64-channel head and neck coil. Image acquisition performed a single-shot T1 multi-echo MPRAGE (voxel size = 1.0 mm isotropic; TR = 2.53 s; TEs = 1.69, 3.55, 5.41, 7.27 ms; TI = 1200 ms; FOV = 256 mm; FA = 7.0; 176 slices, acceleration = 2; readout bandwidth = 650 Hz/Px); T1-weighted FLAIR (voxel size = 1.0 × 1.0 × 1.0 mm; TR = 5 s; TE = 391 ms; FOV = 256 mm; 176 slices; turbo factor = 278); B1 maps (voxel size = 7 × 7 × 14 mm; TR = 9 s; TE = 1.86 ms; FOV = 450 mm; FA = 8.0; 33 slices); SWI (voxel size = 0.9 × 0.9 × 1.8 mm; TR = 30 ms; TE = 20 ms; thickness = 1.79 mm; FOV = 220 mm; FA = 12.0; 80 slices; 3D GRE; acceleration = 2); and T2*-weighted images (voxel size = 1.4 × 1.4 × 3.0 mm; TR = 6 s; TE = 95 ms; echo spacing = 8.64 ms; turbo factor = 21; FOV = 450 mm; FA = 160.0; 31 slices).

The 7 T experiments were performed using a 7 T Siemens Terra scanner with an in-house custom-made 64-channel head coil. We acquired a T1 multi-echo MPRAGE (voxel size = 0.75 mm isotropic; TR = 3.82 s; TE = 1.72, 3.52, 5.32, 7.12 ms; TI = 1140 ms; FOV = 225 mm; FA = 7.0; 224 slices; acceleration = 3; readout bandwidth = 690 Hz/Px) (van der Kouwe *et al* 2008). Functional data were acquired with a BOLD-weighted gradient-echo EPI (voxel size = 1.6 mm isotropic; TR = 1.15 s; TE = 15 ms; FA = 41;; SMS = 2; GRAPPA = 4; FOV = 192 mm; 62 slices; readout bandwidth = 1985 Hz/Px)

### Phantom scans.

The commercial Cu-Net cannot be used on humans with high-SAR sequences due to safety concerns, so phantom scans were used to examine the differences in image quality between the Inknet2 and the Cu-Net. Phantom scans compared three conditions: 1) No-Net, 2) Inknet2, and 3) Cu-Net. Phantom CT scans compared the three conditions with the standard Human Adult Trauma Head 2 mm Bone scan (SOMATOM Force; Siemens Healthineers, Forchheim, Germany). Phantom MRI Scans at 3 T used the same anatomical imaging protocol for each net condition as previously described.

### Human scans—3 Tesla.

We obtained written informed consent and prospectively imaged five healthy volunteers with MRI at 3 T (mean age = 36.2 years; range = 22–66 years; 4 females). We have previously reported preliminary results in an abstract and poster at the *International Society for Magnetic Resonance in Medicine* (Fultz *et al* 2022). Participants underwent one scan session with two conditions in counterbalanced order: 1) No-Net and 2) Inknet2. The Cu-Net was not tested in humans because it is not approved for use with high-SAR sequences, such as the SWI, and thus not safe to scan with a human due to the potential for heating. Each condition followed the same anatomical MR imaging protocol as the phantom scan, including a T1-weighted MEMPRAGE, FLAIR, B1 maps, SWI, and T2*-weighted images.

### Human scans—7 Tesla.

We obtained written informed consent and prospectively imaged 3 healthy volunteers with MRI at 7 T (mean age = 24.33 yrs, age range = 22–26 yr, 2 females). Participants underwent a single 2 h scan session at 7 T with two conditions in counterbalanced order: 1) No-Net and 2) Inknet2. In each condition, participants underwent an anatomical scan and two functional runs. In fMRI runs, participants performed a block design visual task with 16 s ON and 16 s OFF blocks to assess visual responses. ON blocks consisted of a 12 Hz flickering radial checkerboard, and OFF blocks consisted of a median luminance grey screen. The visual flickering checkerboard block design was created using the LÖVE 2D gaming framework software (https://github.com/dangom/love-experiments). Participants were instructed to fixate on a dot placed at the center of the screen. The center dot randomly changed color, and participants were instructed to press a button when the dot changed color to ensure task engagement and maintain fixation.

### Radiologist evaluation.

Anatomical images from the human scans at 3 T were evaluated by two board-certified neuroradiologists (ML and RG). The neuroradiologists were blinded to condition (Inknet2 vs. No-Net) and asked to evaluate the presence of artifacts, overall image quality, and clinical usability with a Likert Scale (supplemental table 1). The T2* sequence was not acquired in one subject. One subject’s FLAIR image was excluded because the subject had severe head motion. In order to blind the neuroradiologists, we excluded all signals outside the brain and skull by masking. A brain mask was generated from the non-brain tissue classes estimated with the brain segmentation and bias field correction algorithm available on SPM (www.fil.ion.ucl.ac.uk/spm/software/spm12/). The automatically created masks were manually examined, and if they still contained visible EEG electrodes (which was the case in three subjects), the FreeSurfer 6.0 erosion tool (https://surfer.nmr.mgh.harvard.edu/fswiki) was used to remove any remaining electrodes. This mask was then applied to acquired anatomical and functional images so that the EEG net was not visible. This EEG removal protocol was then applied to both the Inknet2 and No-Net conditions to ensure consistency across the two conditions (https://github.com/ninafultz/eeg_removal_memprage).

### T1-MEMPRAGE quantitative image analysis.

To examine grey/white matter contrast, the FreeSurfer *pctsurfcon* function was used to compute surface-wise gray/white contrast ratio between the T1-weighted multi-echo MPRAGEs for 34 FreeSurfer ROIs from the Desikan–Killiany atlas (Desikan *et al* 2006). MRI data was processed using FreeSurfer and FSL version 6 (Jenkinson *et al* 2012) (https://fsl.fmrib.ox.ac.uk/fsl/fslwiki). Anatomical images were automatically segmented using FreeSurfer (Fischl *et al* 2002). This quantification was performed on both the 3 T and 7 T datasets.

We then computed the contrast-to-noise ratio (CNR) of grey-to-white matter within the visual cortex and white matter immediately adjacent to the visual cortex where RF shielding is most likely to occur and impact the MRI signal ($\text{CNR}=\frac{\text{SIwhite}-\text{SIgrey}}{\text{SDcombined}}$, where SIwhite and SIgrey are the average signal intensities for the grey and white matter within the visual cortex, respectively, and SDcombined is the standard deviation of the combined grey and white matter signal intensities in the visual cortex). We first extracted masks bilaterally for the lateral-occipital, cuneus, and pericalcarine labels from FreeSurfer’s automatic segmentation. We also extracted the white matter mask and only included voxels immediately adjacent to the visual cortex grey matter mask. CNR was calculated for each subject’s scans with and without the Inknet2 from these masks. A repeated-measures t-test was done to compare the T1-MEMPRAGE grey/white CNR with and without the Inknet2.

### FLAIR quantitative image analysis.

FLAIR sequences typically null out signals from fluid, such as in cerebrospinal fluid (CSF), and has been used to identify microstructural changes in brain tissue (Bahsoun *et al* 2022); thus, we quantified differences in FLAIR signal quality with and without the Inknet2 by computing a CNR between the spaces with CSF and the immediately adjacent cerebral grey matter ($\text{CNR}=\frac{\text{SIgrey}-\text{SICSF}}{\text{SDcombined}}$, where SICSF and SIgrey are the average signal intensities for the CSF and cerebral grey matter, respectively, and SDcombined is the standard deviation of the combined CSF and cerebral grey matter). First, we segmented the FLAIR sequence using the contrast-agnostic tool SynthSeg (Billot *et al* 2023) to obtain regions of interest. Then, we extracted and combined bilateral masks for the lateral ventricles, inferior-lateral ventricles, 3rd ventricle, 4th ventricle, and CSF to create a mask for all CSF spaces. We also extracted the cerebral grey matter mask. Both the CSF and grey matter mask were then eroded to account for voxels mislabeled from SynthSeg and manually reviewed to ensure accuracy. After computing CNR between these two masks, we performed a repeated-measures t-test with and without the Inknet2 to compare.

Second, we calculated white-matter hyperintensities (WMH) within the FLAIR images as this metric is commonly derived from FLAIR sequences and has been associated with several cognitive outcomes with aging (Bahsoun *et al* 2022), as well as cerebrovascular disease in older adults with sleep apnea (Carvalho *et al* 2023). To calculate WMH volume, FLAIR images were processed using a fully automated algorithm (Raghavan *et al* 2021; as implemented in Carvalho *et al* 2023). Briefly, in this automated algorithm (Raghavan *et al* 2021), FLAIR images were preprocessed for intensity inhomogeneity correction (Zhang *et al* 2001) and were de-noised using a non-local means filter (Manjón *et al* 2010). White matter was then segmented using SynthSeg (Billot *et al* 2023) to segment these regions of interest with this contrast-agnostic tool. False-positive WMH segmentations were reduced by applying a white matter mask derived from the T1-MEMPRAGE segmentation. WMH was finally adjusted for total intracranial volume (TIV). Therefore, WMH is measured as a percentage of TIV. TIV was taken from FreeSurfer’s recon-all statistics output. A repeated-measures t-test on WMH measures with and without the Inknet2 was computed to compare the two conditions.

### SWI quantitative image analysis.

SWI is sensitive to compounds that distort the local magnetic field, such as calcium and iron, and is a valuable tool for visualizing venous structure and iron in the brain (Halefoglu and Yousem 2018). To compare the quality of images with and without the Inknet2 cap, we measured the overlap of voxel intensities by computing the Dice coefficient ($\text{Dice}=\frac{2*|X\cap Y|}{\left|X|+|Y\right|}$, where *X* is the SWI magnitude image without the Inknet2 cap, and *Y* is the SWI magnitude image with the Inknet2 cap). Dice coefficients range between 0 and 1, where 1 indicates complete overlap between images; thus, a Dice coefficient close to 1 indicates good overlap and few differences in voxel magnitude. To compute the Dice coefficient, we first extracted the brain mask within the SWI image using mri_synthstrip (Hoopes *et al* 2022). Then we co-registered the two images together using rigid robust registration (Reuter *et al* 2010) before computing the Dice coefficient between the two images per subject.

### T2* quantitative image analysis.

The T2*-weighted acquisition, with a long TR and short TE, results in hyperintense CSF and attenuates the signal in grey and white matter. To compare the T2* image quality with and without the Inknet2, we computed the CNR between the CSF spaces and the immediately adjacent white matter regions, similar to the computation for the FLAIR sequence above ($\text{CNR}=\frac{\text{SICSF}-\text{SIgrey}}{\text{SDcombined}}$, where SICSF and SIgrey are the average signal intensities for the CSF and cerebral grey matter, respectively, and SDcombined is the standard deviation of the combined CSF and cerebral grey matter). First, we segmented the T2* sequence using SynthSeg (Billot *et al* 2023). Then, we extracted and combined bilateral masks for the lateral ventricles, inferior-lateral ventricles, 3rd ventricle, and CSF to create a mask for all CSF spaces. Note that the T2* sequence coverage does not cover most of the brainstem. Thus, the 4th ventricle is not included in this CSF mask. We also extracted the cerebral grey matter mask. After computing CNR between these two masks, we performed a repeated-measures t-test with and without the Inknet2 to compare.

### Additional quantitative image analysis across modalities.

Traditional image quality metrics were also computed to further assess differences across anatomical scans with and without the Inknet2. Prior to computing any of the following metrics, mri_synthstrip was used to extract the brain from each anatomical image (Hoopes *et al* 2022). Then, each skull-stripped anatomical image acquired with the Inknet2 cap was co-registered with the equivalent image acquired without the Inknet2 cap using rigid robust registration (Reuter *et al* 2010). The first metric computed was a structural similarity index (SSIM), which is a common metric used to assess the similarity of two images by taking into account differences in structural information, luminance, and contrast (Wang *et al* 2004, Qasim *et al* 2018, Sara *et al* 2019, Guerreiro *et al* 2023). SSIM values range from −1 to 1, where 1 indicates perfect similarity between two images and 0 indicates no similarity (Wang *et al* 2004). Second, we computed a feature similarity index (FSIM), which is an image quality assessment metric that evaluates the similarity between two images based on their features, such as phase congruency and gradient magnitude of the images (Zhang *et al* 2011). FSIM has normalized values where 1 indicates perfect similarity and 0 indicates no similarity between images. SSIM and FSIM were specifically designed to be more sensitive to both local and global details of the image, making them useful measures for identifying changes in anatomical image quality with the Inknet2 (Sara *et al* 2019).

### B1.

Maps were visually inspected for field homogeneity and compared between conditions to assess image quality.

### fMRI preprocessing and statistical analysis.

Functional images at 7 T were slice-time corrected using FreeSurfer (Fischl 2012) and motion-corrected using AFNI (Cox and Jesmanowicz 1999). Images were then registered to the anatomical MPRAGE using *bbregister* (Greve and Fischl 2009). FEAT (FMRI Expert Analysis Tool; version 6.00) was used to extract the *z*-score of functional responses from the visual-stimulation block design based on the timing of the visual stimulus convolved with a canonical hemodynamic response function, and the contrast between No-Net and Inknet2 conditions was calculated to test for any differences between No-Net vs. Inknet2 conditions for each subject (Worsley 2001). Cluster correction was done with a *z*-value threshold of 3.1 and a corrected cluster significance threshold of *p* = 0.05.

## 3. Results

### The Inknet2 design and effect on image quality.

**Figure 2.**
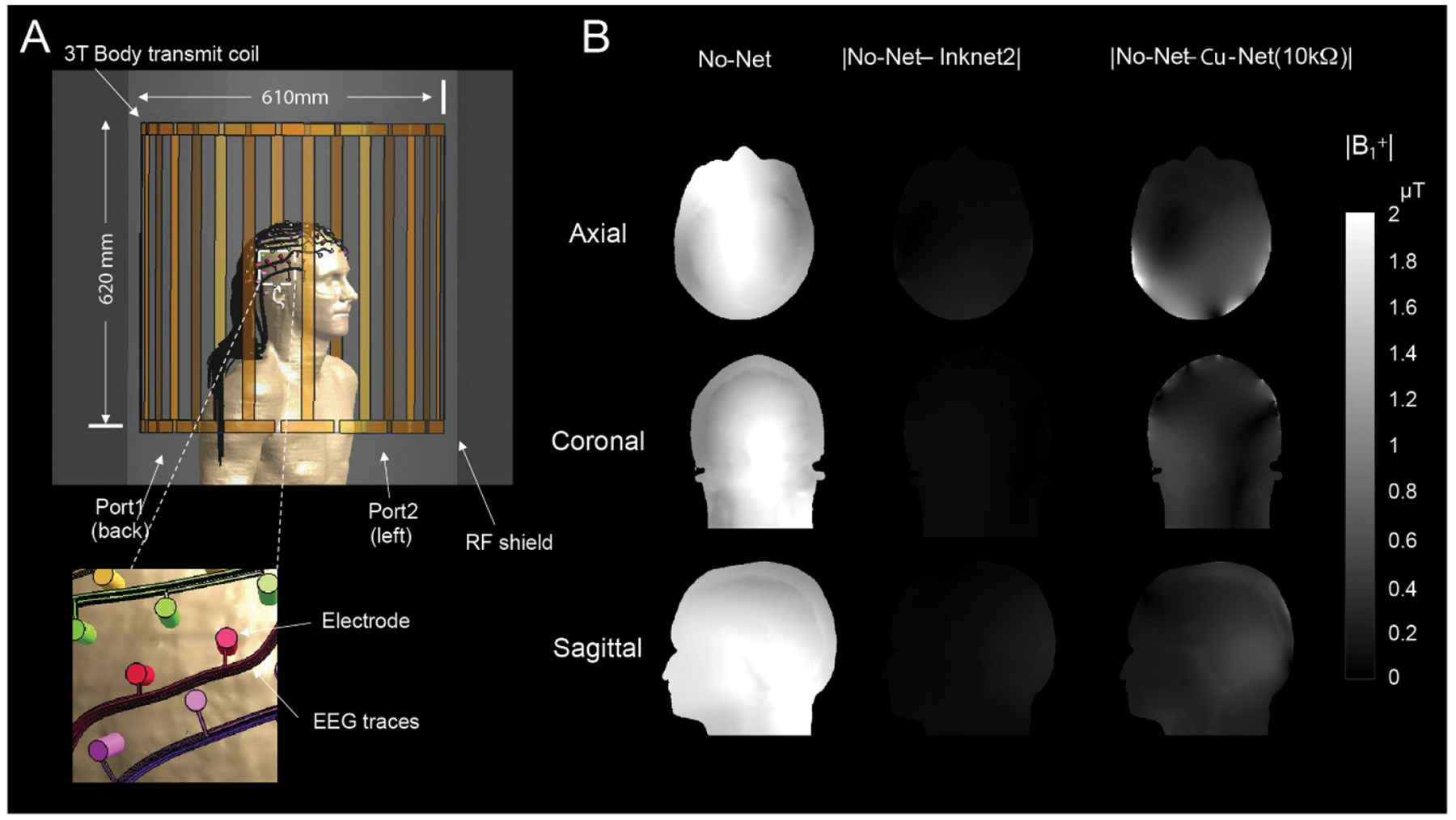
Numerical simulation shows image quality improvements with Inknet2. (A) 128-channel EEG net on the Duke model positioned in the center of the body transmit coil tuned at 3 T (fields were normalized to 2 *μ*T at the center of the coil). (B) B1 transmitting field maps are displayed in the case of No-Net in axial, coronal, and sagittal views, and the difference in the ${\text{B}}_{1}^{+}$ field between No-Net vs. Inknet2 and No-Net vs. Cu-Net is shown.

We constructed the Inknet2 using the same PTF technology as in prior work (Poulsen *et al* 2017) but with a new modular design in which each trace was printed separately (one electrode per PTF trace), which allows for easier manufacturing as well as flexible design of electrode layouts to construct caps for multiple head sizes. We performed safety tests to confirm no substantial heating with this new design (supplemental figure 2) and then used simulations to model the effect of this cap design on image quality. Numerical simulations showed that the $B_{1}^{+}$ field artifact volume using the Inknet2 was reduced compared to the Cu-Net (figure 2). The maximum difference in the $B_{1}^{+}$ field was estimated as 0.43 *μ*T for the No-Net vs. Inknet2 comparison and 3.16 *μ*T for the No-Net vs. Cu-Net comparison. The root-mean-squared error in the $B_{1}^{+}$ field was 0.09 *μ*T for No-Net vs. Inknet2 and 0.29 *μ*T for No-Net vs. Cu-Net (figure 2). These simulations suggested that the Inknet2 would have improved image quality compared to conventional nets.

### Phantom scans.

**Figure 3.**
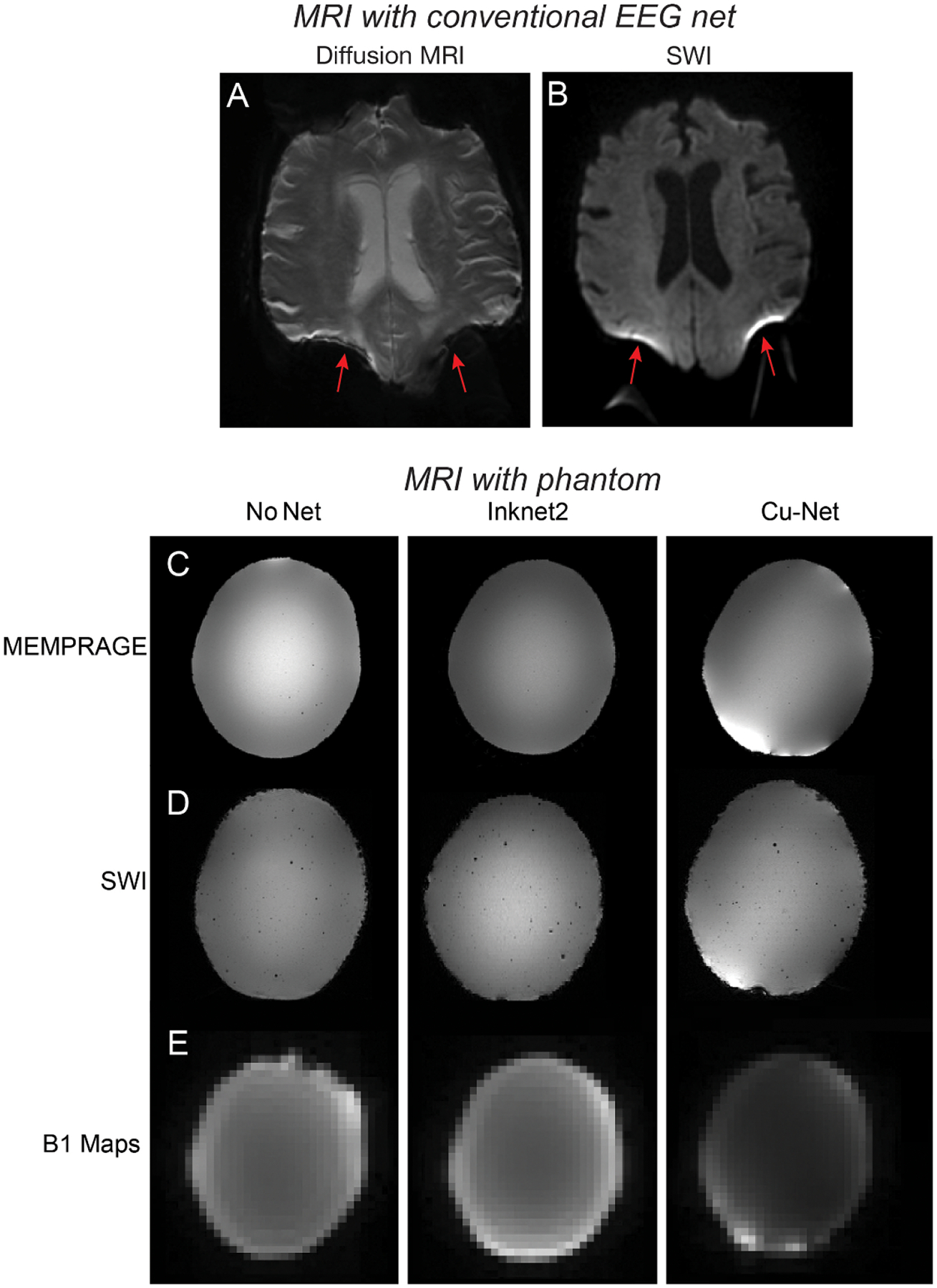
MR images exhibit artifacts when conventional EEG electrodes are present. (A), (B) Clinically acquired MR images in patients undergoing routine clinical imaging show artifacts if EEG electrodes are not removed prior to imaging. (A) Diffusion-weighted MR images were severely degraded posteriorly by artifacts (red arrows) from EEG electrodes. (B) Artifacts (red arrows) from EEG electrodes severely degraded SWI MR images. (C)–(E) Comparison of No-Net, Inknet2, and Cu-Net on a phantom. Anatomical images from the Inknet and Cu-Net conditions are displayed with the electrodes manually masked out. All anatomical images have identical grey scales across conditions. (C) MEMPRAGE shows similar image quality in the Inknet2 and No-Net conditions and a decrease in image quality in the Cu-Net condition, seen as focal high intensity. (D) SWI maps show increased dropout in Cu-Net conditions and increased artifact from the individual electrodes on the Cu-Net. (E). The B1 field map shows similar radiofrequency field quality in No-Net vs. Inknet2 conditions but a reduction in the Cu-Net condition.

Clinically acquired MRI images with conventional EEG electrodes show severely degraded image quality (figures 3(a) and (b)). The commercial Cu-Net cannot be used on humans with high SAR MRI sequences, thus we used phantom scans to test the Inknet2 performance in the MRI environment based on the positive simulation results. The MEMPRAGE and SWI both showed focal dropout in the Cu-Net condition and substantial changes in image intensity in the posterior region of the phantom, whereas this issue was not present with the Inknet2 (figures 3(c) and (d)). The B1 field maps showed a large difference between the Cu-Net and the Inknet2, whereas there was no clear difference between the Inknet2 and the No-Net condition (figure 3(e)), suggesting an effective reduction of the RF shielding caused by conventional EEG nets. Overall, our phantom scans showed a clear improvement of the Inknet2 on gross image quality compared to the conventional Cu-Net.

Since CT imaging is also widely used in clinical practice, and the ability to perform a high-quality CT scan in ICU patients without pausing to remove an EEG net could be of high clinical benefit, we additionally qualitatively evaluated image quality on the phantom in the CT scanner. A qualitative comparison of the phantom CT images showed that the Cu-Net condition had substantial artifacts from the copper wires, which impeded visualization of the CT image (supplemental figure 1). While the Inknet2 electrodes were also visible, no artifacts from the leads were visible (supplemental figures 1(b) and (c)).

### Anatomical MRI quality with the Inknet2.

**Figure 4.**
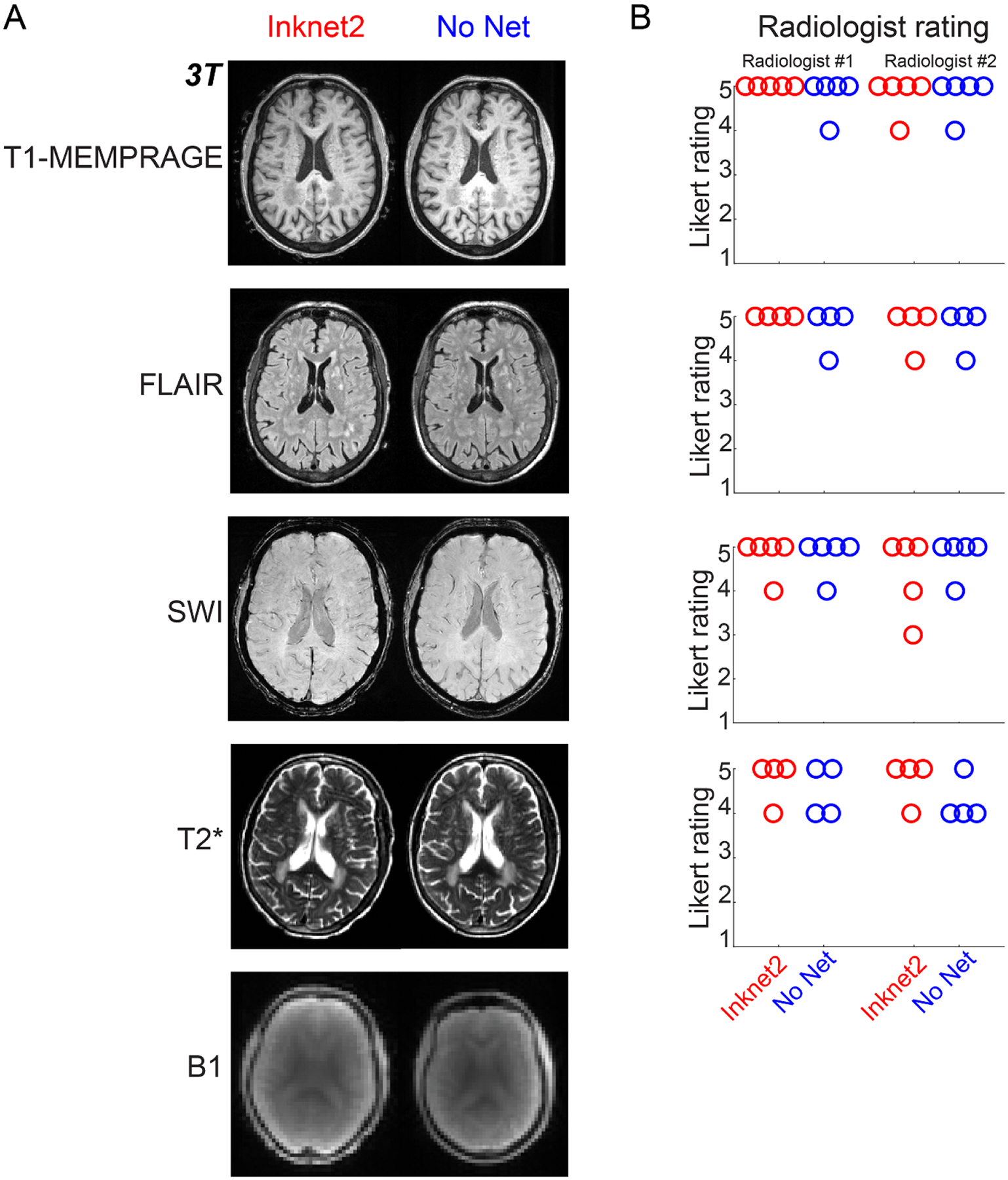
Human comparison of No-Net vs. Inknet2 conditions with 3 T anatomical scans (which could not be repeated with the Cu-Net condition because of safety constraints). All anatomical images have identical color scales between the Inknet2 and No Net images. (A) 3 T T1-MEMPRAGE, FLAIR, SWI, and T2* images each show similar image quality and contrast across conditions. B1 Field maps show similar radiofrequency fields in both conditions. (B) Image quality ratings from blinded board-certified neuroradiologists for the 3 T anatomical scans indicated similar clinical usability with both conditions for each image type. Each dot represents the Likert scale (see supplemental table 1) rating by one radiologist for one subject. Images were manually masked to remove the EEG electrodes outside the skull to maintain blinding to the condition. Additional slices for all modalities are shown in supplemental figures 5–9.

We next examined image quality with and without the Inknet2 in human subjects based on the promising results from simulations and phantom scans. The human data also showed qualitatively good image quality, with minimal perceptible artifacts across each sequence tested and across magnetic field strengths (figure 4 and supplemental figures 5–9). By contrast, this imaging protocol could not even be acquired with the Cu-Net in humans for comparison since conventional EEG nets pose a safety concern with high-SAR imaging. Image quality and contrast were qualitatively similar across conditions in each scan type, with no obvious artifacts. To validate this finding further, two board-certified neuroradiologists assessed image quality while blinded to condition, and these clinical ratings showed similar image quality in the Inknet2 and No-Net conditions (figure 4).

To quantitatively assess the utility of the Inknet2 at 3 T, we applied commonly used tools for quantifying anatomical and functional properties. We found no overall change in the effectiveness of automated anatomical segmentation with a qualitative comparison of pial and white matter surfaces (figure 6(a)). Quantitative evaluation of grey-white matter mean signal intensity ratios from the T1-MEMPRAGE showed no significant difference between No-Net and Inknet2 conditions, suggesting a preserved whole-brain grey-white matter contrast with the Inknet2 (*t*(33) = 1.15, *p* = 0.256) (figure 6(a)). Since EEG wires bundle towards the back of the head, RF shielding is most likely to occur and impact the MRI signal in posterior regions (Luo and Glover 2011); thus, we further quantified CNR in the visual cortex and nearby posterior regions with and without the EEG cap on. Measuring the CNR between posterior white-grey matter in T1-MEMPRAGE showed no significant difference between conditions, indicating that Inknet2’s PTF technology reduces artifacts near the EEG net’s wire bundle (*t*(4) = −0.375, *p* = 0.726) (figure 6(a)).

In the FLAIR sequence, CNR between grey matter and CSF showed no significant difference between conditions (*t*(3) = −2.11, *p* = 0.125), indicating that the Inknet2 preserved the contrast between grey matter and attenuated fluids in FLAIR scans (figure 6(b)). An additional metric commonly derived from FLAIR sequences, WMH volume normalized by TIV, was also preserved when the Inknet2 was present (*t*(3) = 0.983, *p* = 0.398). The Dice coefficients for SWI magnitude images indicated a high degree of overlap, indicating a similar magnitude signal across the SWI image with and without the Inknet2 (mean = 0.886, sd = 0.051). Difference maps between SWI with and without the Inknet2 also indicated similar image contrast (figure 6(d)). T2*-weighted images showed no significant difference between the subject’s CSF-cerebral gray matter CNR in the T2* sequence, suggesting that the contrast between the grey matter and fluid spaces was preserved with the Inknet2 (*t*(3) = −0.615, *p* = 0.581) (figure 6(c)). Overall, the anatomical metrics specific to each image contrast demonstrated that the Inknet2 yielded image contrast and quantification similar to the No-Net condition, pointing to preserved image quality with the Inknet2 across a range of pulse sequences. Importantly, these comparisons could not be made with the conventional Cu-Net since it is not approved for use with these image modalities, demonstrating that Inknet2 provides the first method for acquiring diverse MRI contrasts in the presence of an EEG net.

**Figure 5.**
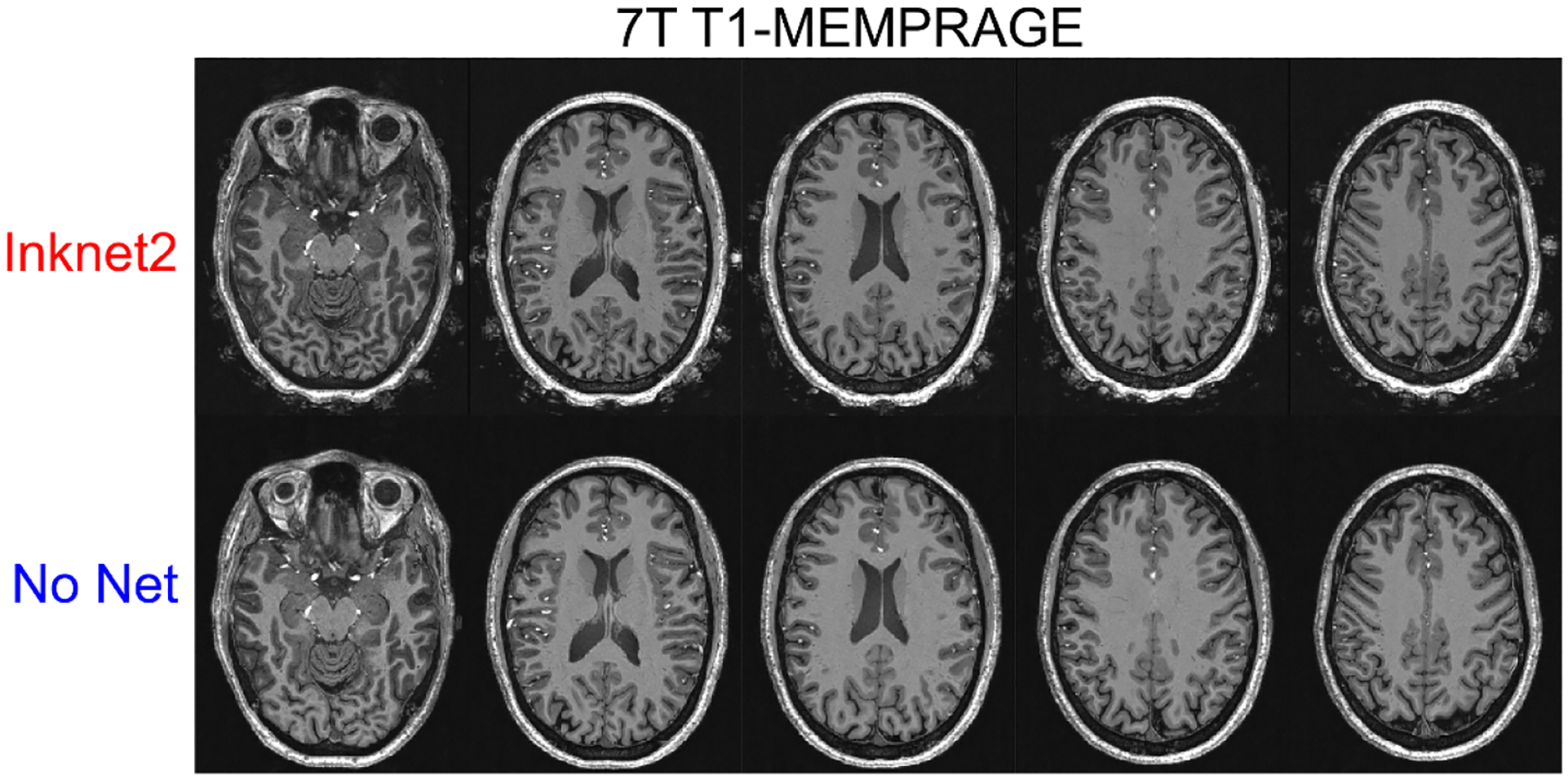
Human comparison of No-Net vs. Inknet2 conditions with 7 T T1-MEMPRAGE. All anatomical images have identical color scales between the Inknet2 and No Net images. 7 T T1-MEMPRAGE shows similar image quality and contrast across conditions.

Next, we performed human studies at 7 T to compare anatomical and functional image performance at high spatial resolution, focusing on the most common sequences used for EEG-fMRI (T1 and gradient-echo EPI). Similar to the 3 T results, quantitative evaluation of grey/white matter mean signal intensity ratios showed no significant difference between No-Net and Inknet2 conditions, indicating that the Inknet2 preserves anatomical image quality at ultrahigh magnetic fields (*t*(33) = 0.826, *p* = 0.414) (figure 5(a)).

**Figure 6.**
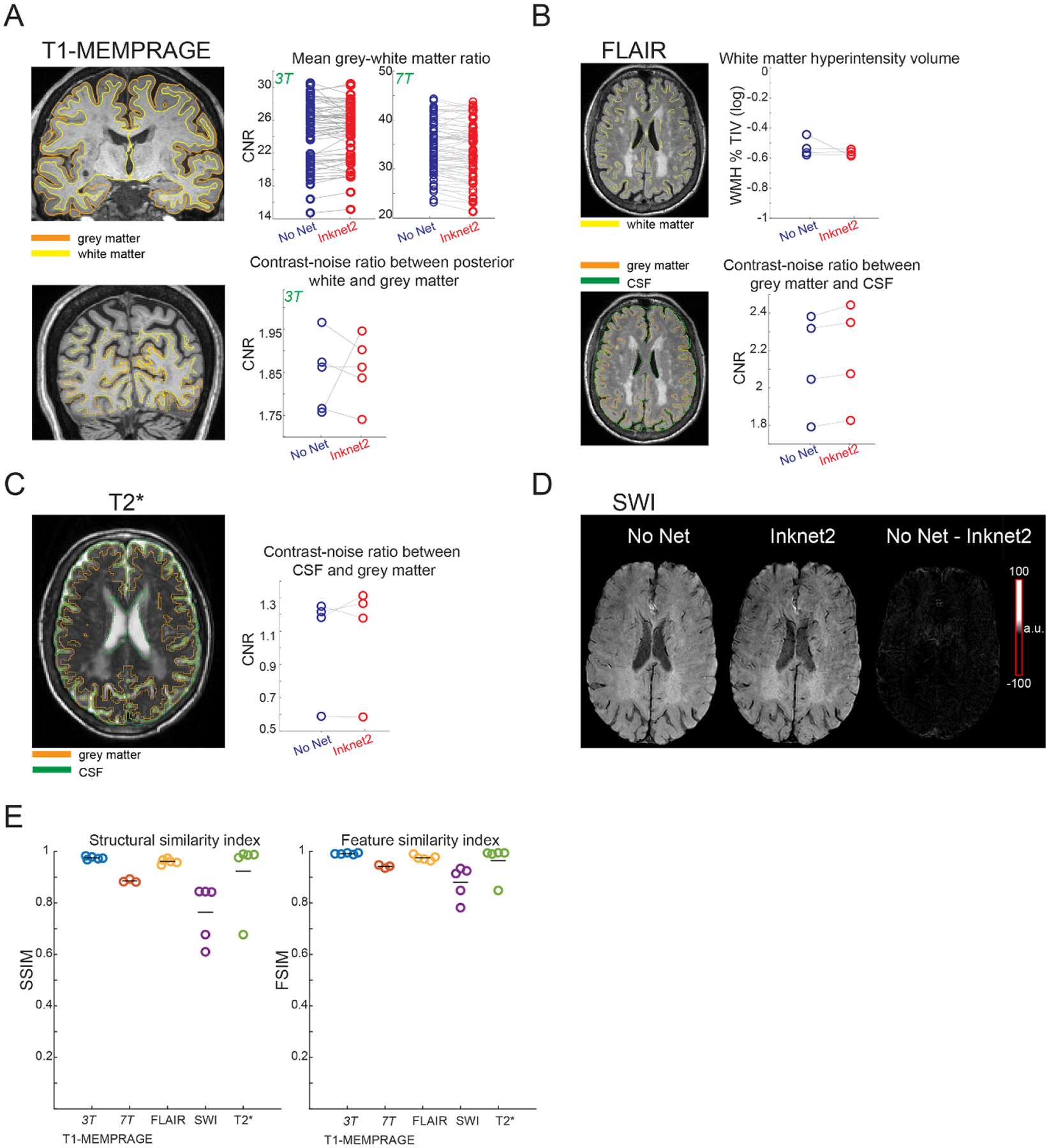
Anatomical contrast is preserved with the Inknet2. (A) Qualitative comparison of pial and white matter surfaces generated by automated FreeSurfer reconstructions, showing similar performance across conditions. The top panel shows the mean grey-white matter ratio for all subjects across 34 cortical regions and shows no significant difference between conditions for both 3 Tesla (*t*(33) = 1.15, *p* = 0.256) and 7 Tesla cohorts (*t*(33) = 0.826, *p* = 0.414). The bottom panel shows the CNR between posterior white-grey matter in T1-MEMPRAGE at 3 T, which indicated no significant difference between the subject’s posterior white-grey matter CNR (*t*(4) = − 0.375, *p* = 0.726). (B) FLAIR images show no significant difference in CNR between grey matter and CSF (*t*(3) = − 2.11, *p* = 0.125). WMH volume normalized by TIV also indicated no significant difference (*t*(3) = 0.983, *p* = 0.398). (C) T2* images showed no significant difference between the subject’s CSF-cerebral gray matter CNR (*t*(3) = − 0.615, *p* = 0.581). (D) SWI magnitude images indicated good overlap with minor differences between the two conditions. (E) Image quality metrics (FSIM and SSIM) across the Inknet2 and No-Net condition. Each dot indicates a single subject. Black lines indicate the mean value across scans. SSIM = structural similarity index; FSIM = feature similarity index.

Finally, to quantitatively compare image quality between all sequences with the same metric, we calculated traditional image processing metrics to quantify similarity between the No-Net and Inknet2 conditions. We found that the SSIM, which captures differences in luminance, contrast, and structural information between two images, indicated close image similarity between scans with and without the Inknet2, as values for all scans were close to 1 (3 T T1-MEMPRAGE: mean = 0.97, sd = 0.005; 7 T T1-MEMPRAGE: mean = 0.89, sd = 0.006; FLAIR: mean = 0.96, sd = 0.01; SWI: mean = 0.76, sd = 0.11; T2*: mean = 0.92, sd = 0.13) (figure 6(e)). As a second metric, we also examined the FSIM. While SSIM is sensitive to local changes in luminance, contrast, and structural information in images, FSIM is instead sensitive to feature changes, such as phase congruency and gradient magnitude, assessing different aspects of image similarity than SSIM (Sara *et al* 2019). We found that the FSIM also indicated strong similarity between images with and without the Inknet2 (3 T T1-MEMPRAGE: mean = 0.99, sd = 0.003; 7 T T1-MEMPRAGE: mean = 0.94, sd = 0.006; FLAIR: mean = 0.97, sd = 0.009; SWI: mean = 0.88, sd = 0.06; T2*: mean = 0.96, sd = 0.06) (figure 6(e)). The SWI scan exhibited the lowest scores with both metrics, suggesting minor differences in image quality when using the Inknet2; however, the overall high value nevertheless indicated a largely preserved image between the Inknet2. As SSIM and FSIM take into account information about image structure and features, these results demonstrated strongly preserved image quality with and without the Inknet2 across all sequences.

### fMRI quality with the Inknet2.

**Figure 7.**
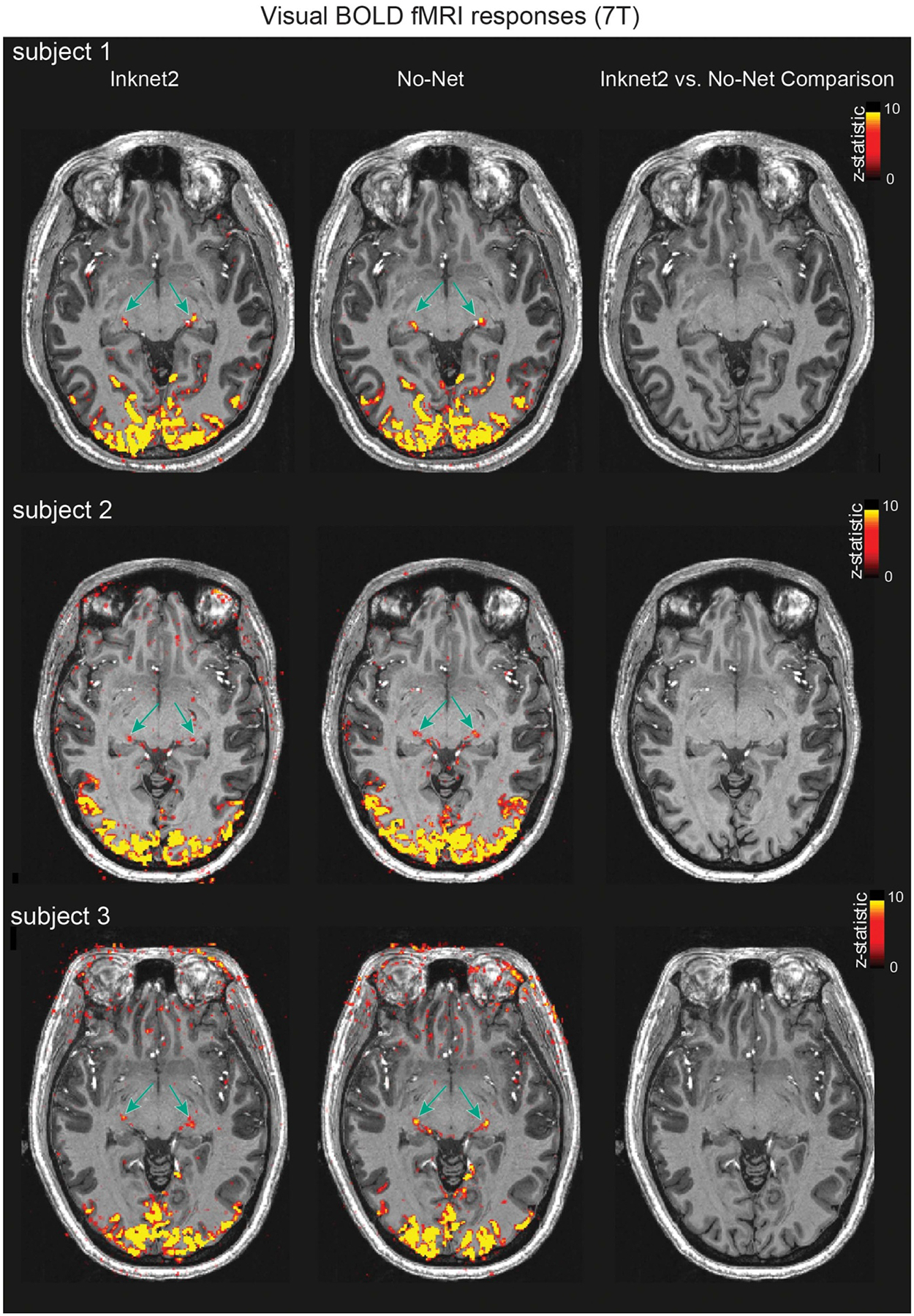
Functional responses are not significantly different with the Inknet2 cap. Visual-evoked BOLD fMRI responses show no consistent difference between No-Net and Inknet2 at 7 T for each subject (*p* > 0.05). fMRI responses were clearly detected in each condition per subject, including in challenging regions such as the small focal activations bilaterally in the lateral geniculate nucleus of the thalamus (green arrows). The color indicates voxel-wise multiple-comparisons-corrected *z*-statistic values (thresholded at *z*stat > 3.1) overlaid on an anatomical image. Negative voxels are not shown.

We next examined how the Inknet2 affected functional signals, analyzing the BOLD response at 7 T to a flickering checkerboard visual stimulus across No-Net and Inknet2 conditions. The visual stimulation task produced strong visuocortical and visual thalamic activations, both with and without the Inknet2 (figure 7). Comparison across conditions within each subject indicated no consistent differences in functional activation with and without the Inknet2 (all voxels *p* > 0.05, multiple-comparisons corrected voxelwise). Furthermore, responses even in low-SNR regions such as individual thalamic nuclei, such as the lateral geniculate nucleus, were still clearly detected in the Inknet2 condition, using just two runs for each subject (figure 7, green arrows). These results indicate broadly preserved visual fMRI responses across the visual cortex and visual thalamus with the Inknet2.

## 4. Discussion

Our results demonstrate that a PTF-based EEG cap enables a wide range of anatomical and fMRI sequences to be performed in the presence of EEG electrodes at both 3 T and 7 T without sacrificing image quality. Both phantom and human scans demonstrated that the Inknet2 enables the acquisition of diverse MRI contrasts in the presence of an EEG net. This technology thus provides EEG traces compatible with high-power MR imaging sequences and ultra-high field scanners, opening the door to new types of multimodal imaging studies.

Our quantitative comparison of anatomical images acquired with and without the Inknet2 demonstrated high image quality when scanning with the Inknet2. The scan-specific metrics, analyzing the outcome of important analyses such as segmentation and feature quantification, showed similar results with and without the Inknet2. In addition, traditional image metrics directly comparing anatomical images with and without the Inknet2 found overall preserved image quality even with the Inknet2. The SWI scan exhibited the lowest SSIM and FSIM values and a larger standard deviation, suggesting that small effects do exist for this sequence type. This finding is likely a result of inhomogeneities in the magnetic field introduced by the presence of the Inknet2, which SWI is particularly sensitive to (Halefoglu and Yousem 2018). Although SWI on average exhibited somewhat lower structural image similarity compared to the other modalities tested, the SSIM and FSIM values for SWI images with and without the Inknet2 still suggested largely preserved image structure and feature information with the Inknet2. As SSIM and FSIM have been suggested to capture both global and local aspects of image quality (Wang *et al* 2004, Qasim *et al* 2018, Sara *et al* 2019, Guerreiro *et al* 2023), our results indicate high structural and feature similarity across images with and without the Inknet2.

This method has potential applications in a wide range of research areas, as it enables EEG to be acquired with multimodal MRI measurements that have not previously been combined with EEG. For example, diffusion contrasts have been widely explored as a readout of rich anatomical and potentially functional information about the water movement in brain and tissue microstructure (Schaefer *et al* 2000, Iturria-Medina *et al* 2007); however, to date, diffusion has not been combined with simultaneous EEG due to the high-SAR nature of typical protocols. The inability to track other aspects of neural properties strongly limits the types of neuroscience studies that can be performed. For example, EEG-fMRI studies to date have nearly all been performed with gradient-echo EPI, measuring the BOLD fMRI signal, which has been powerful for identifying coupling between neural activity and hemodynamic responses (Dang-Vi *et al* 2008, Morillon *et al* 2010, Dresler *et al* 2012, Walz *et al* 2013, Marawar *et al* 2017, Fang *et al* 2019, Fultz *et al* 2019, Setzer *et al* 2022). However, when fMRI was adapted to measure a distinct functional contrast—the flow of CSF—it unexpectedly revealed that EEG activity is also coupled to fluid flow in the brain (Fultz *et al* 2019). As the Inknet2 technology makes diffusion, susceptibility, and diverse structural contrasts possible with simultaneous EEG, future studies can now examine how other unexpected features of brain function and structure are related to the rapid dynamics of EEG fluctuations.

In addition to expanding the scientific applications of multimodal imaging, PTF-based EEG net technologies could be valuable in clinical settings. Continuous EEG is widely utilized in critically ill patients to detect seizures and other neurophysiological events, and to inform rapid clinical intervention (Ney *et al* 2013). Since these patients frequently also need MRI imaging for their clinical care, PTF-based nets would be valuable for allowing continuous EEG monitoring without interruptions for clinical imaging. In addition, a new wave of engineering advances is beginning to enable portable MRI, aiming to provide anatomical imaging at the bedside and in point-of-care settings (Wald *et al* 2020, Cooley *et al* 2021, Mazurek *et al* 2021). Integrating PTF net technology could in the future enable easy use of multimodal EEG-MRI in this wide range of settings.

This study includes several limitations. A comparison of EEG nets in the phantom and human scans required the subject to be removed from the scanner bore in order to place the new EEG net, resulting in moderately different head orientations across conditions. We accounted for this by adjusting pillow placement and asking subjects to keep a consistent position throughout the scan. In addition, the conventional EEG nets (Cu-Net) could not be assessed in human imaging due to safety concerns; however, numerical simulations and phantom results clearly indicated worse image quality with the Cu-Net, suggesting that even if safe for human use, the Cu-Net would still suffer from reduced image quality compared to the Inknet2. Additionally, our small sample size was designed to provide proof-of-concept and test for large effects on image quality. The Inknet2 may still cause subtle changes in image quality that were not detected in the current study, which could be identified in a larger sample. However, our results nevertheless demonstrate a proof-of-concept for successful imaging, as we found no major change in the effectiveness of automated anatomical analysis approaches and no clear artifacts when the Inknet2 was present. Also, the full set of pulse sequences tested at 3 T were not also tested at 7 T due to the limited time per session, leading us to focus on EPI and T1, since they are currently commonly used sequences in 7 T imaging. Future work could expand into additional sequence types at 7 T as well, particularly as clinical imaging at 7 T becomes more widespread. Finally, a significant limitation is that our measurements were performed using a single instantiation of this EEG hardware; safety and image quality must still be established at local sites with their own equipment or via a standardized process if nets using this technology become commercially manufactured in the future.

Expanding access to Inknet2 in the future is a key next step to enable a range of multimodal imaging studies with these diverse sequences. Since the layout and hardware of different MRI scanners can vary substantially (Shinohara *et al* 2014, Fortin *et al* 2016, Wittens *et al* 2021, Kushol *et al* 2023), a useful next step would be to test its performance in other scanner systems. Furthermore, the use of reference-based EEG noise removal methods is highly effective for improving EEG signal quality in EEG-MRI (Luo *et al* 2014, van der Meer *et al* 2016, Levitt *et al* 2023). Adding noise measurement devices, such as carbon wire loops, to the Inknet2 could further enhance its ability to provide high-quality EEG signals, particularly at ultra-high magnetic field where EEG artifacts become larger. Finally, while the traces can be readily manufactured by printing companies, assembling them into a functional net is a more labor-intensive process, so future work will need to develop either a larger-scale manufacturing process or training courses on how to build these nets. With these updates, the Inknet2 could offer a unique set of capabilities for researchers to perform multimodal EEG-MRI across field strengths and sequences.

## 5. Conclusion

Our results demonstrate that high-resistance-lead PTF technology enables EEG nets to be combined with a wide array of MRI protocols without compromising image integrity. This technology could thus allow for simultaneous EEG-MRI with a broad range of sequences at 3 T and 7 T, allowing new forms of multimodal imaging and providing rich information about brain function and structure.

## Supplementary Material

Supplementary Materials

## Data Availability

The data that support the findings of this study are openly available at the following URL/DOI: https://doi.org/10.18112/openneuro.ds005533.v1.0.0 (Cicero *et al* 2024). Clinical data that was analyzed under a secondary-use IRB (figures 3(a) and (b)) cannot be publicly uploaded due to privacy and IRB regulations. Data analysis used standard and publicly available toolboxes.
