# Supplementary material for: High-quality multimodal MRI with simultaneous EEG using conductive ink and polymer-thick film nets
Source: J Neural Eng. Author manuscript; Available in PMC 2025 Jan 14. (PMC11732253; doi:10.1088/1741-2552/ad8837)
Supplement: Supplementary Materials [file NIHMS2046398-supplement-Supplementary_Materials.docx]

**Supplementary Figures and Table:**

**
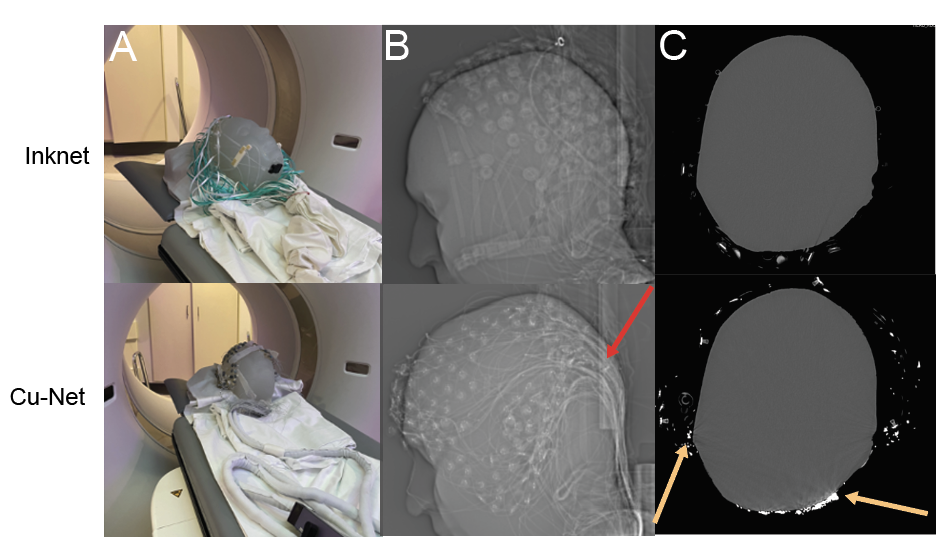
Supplemental Figure 1: Inknet2 with CT imaging in a phantom.** A) Experimental layout of the Inknet2 and Cu-Net in the CT scanner on agar phantom. B) Inknet2 in CT scan has no artifact from EEG leads, whereas Cu-Net CT image indicates significant artifact from wires (red arrow). C) CT localizer shows little artifact presence in the outer cortex with Inknet2 and a significant presence of artifact in the outer cortex with Cu-Net (orange arrows).

**Supplemental Figure 2: 3T MRI RF safety test results of Inknet2 with an adult-sized head agar phantom.** A) The temperature elevation with a high-power turbo spin-echo sequence with the maximum allowed input power in clinical scan for 30 minutes using a birdcage body transmit coil. B) The spatial layout of the 128-channel EEG traces, highlighting the position of EEG electrodes where the temperature was monitored. C) Table of temperature measured on the phantom at timepoint 0 (prior to scanning) and then at the end of the scan (1828 seconds).

**
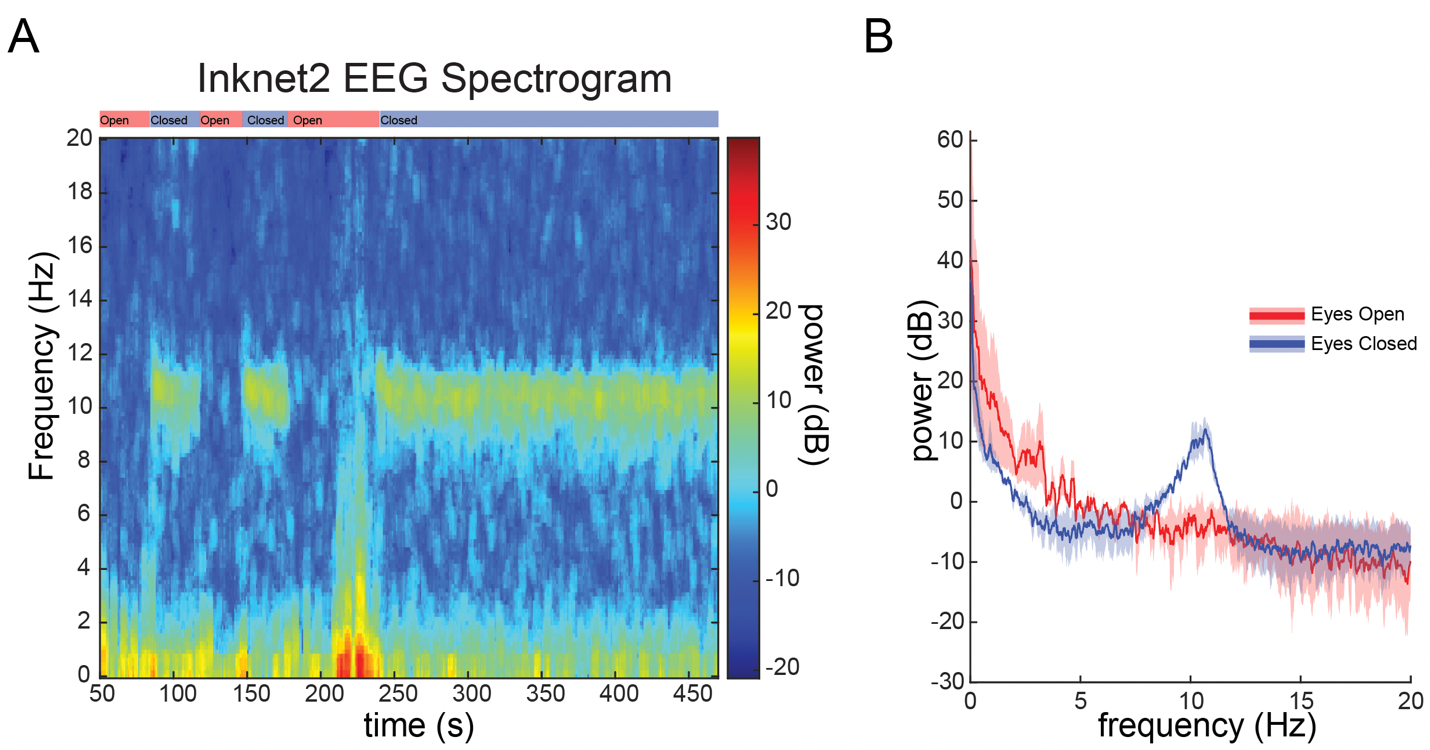
Supplemental Figure 3: Inknet2 provides good EEG quality.** A) EEG spectrogram from an occipital electrode outside of the scanner indicates typical EEG quality when subjects open and close their eyes. During periods of eye closure (blue bars), there is a clear increase in alpha (8-12 Hz) power, compared to periods of eye opening (red bars). B) Average EEG spectrum from occipital electrode during periods of eyes open and eyes closed shows increase in alpha (8-12 Hz) power during eye closure. Shading is standard error.

**
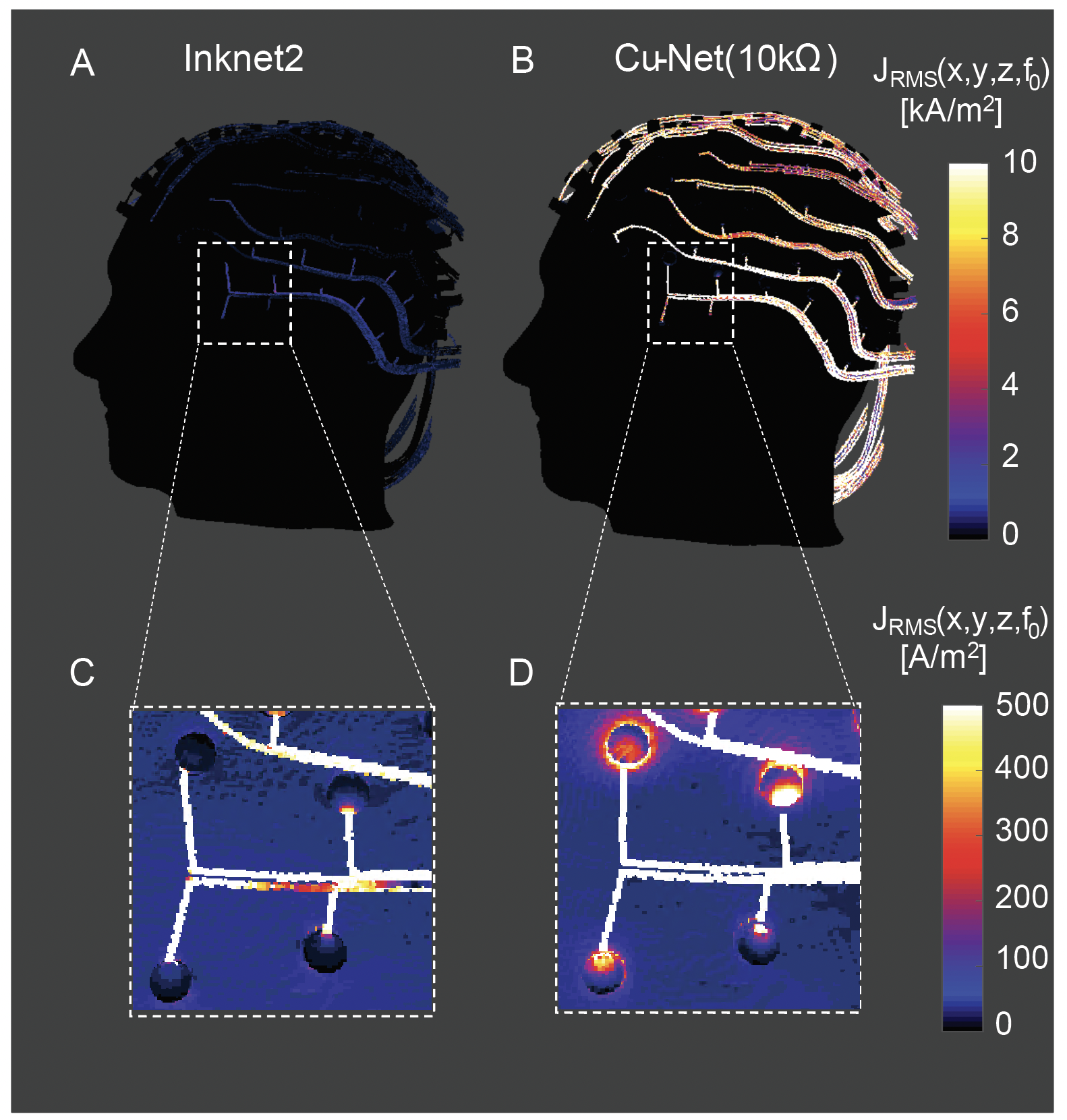
Supplemental Figure 4: Current density map displayed on EEG traces in 3D surface view.** A) Inknet2 B) Cu-Net with ideal current limiting resistors (10kΩ) C) Zoomed view of current density maps in the case of Inknet2 D) Zoomed view of current density maps in the case of Cu-Net with ideal current limiting resistors (10kΩ). The panel shows regions of high density in the Cu-Net that are no longer present in the Inknet2.

**
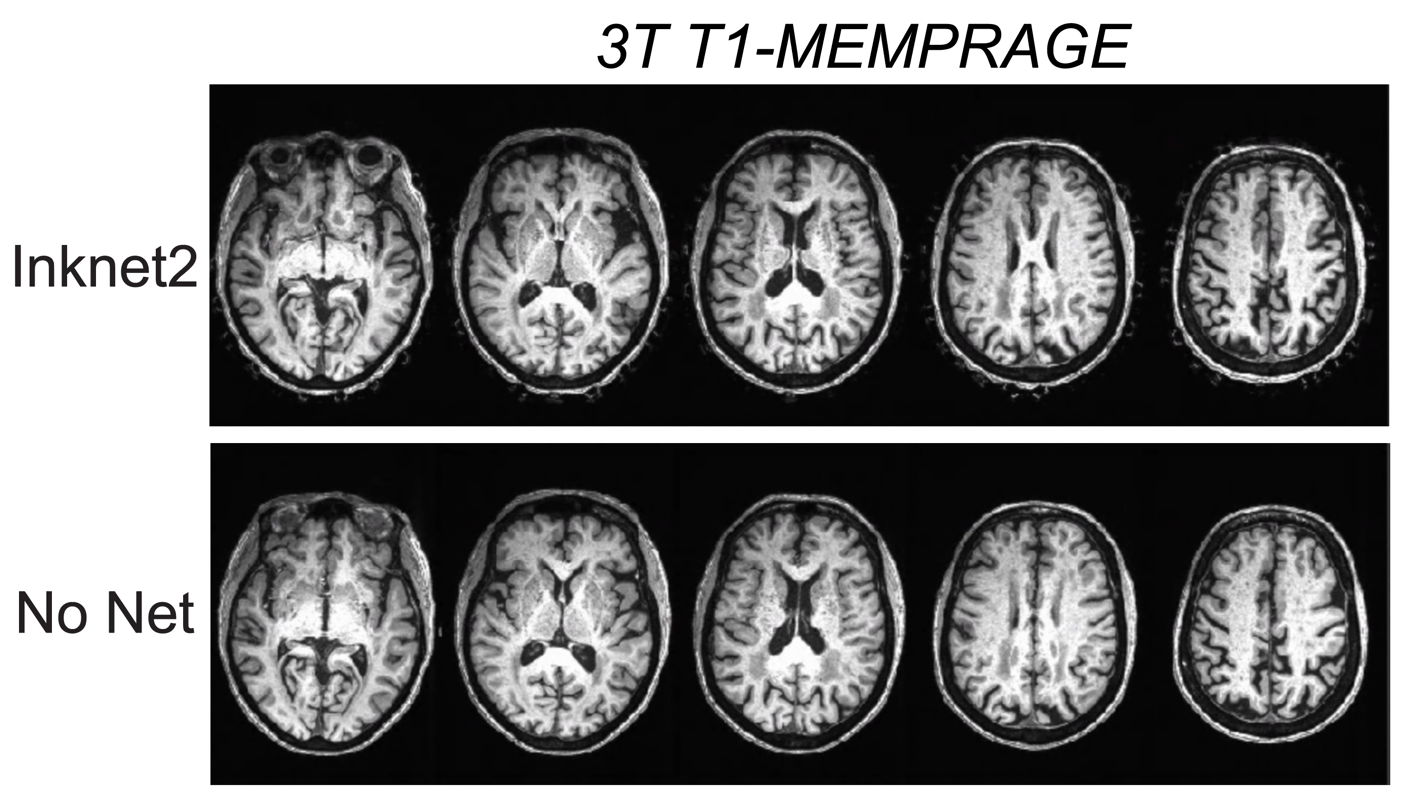
Supplemental Figure 5: Image quality and contrast are preserved in the 3T T1-MEMPRAGE with the Inknet2.** All images have identical color scales. Images were manually masked to remove the EEG electrodes outside the skull to maintain blinding to the condition.

**
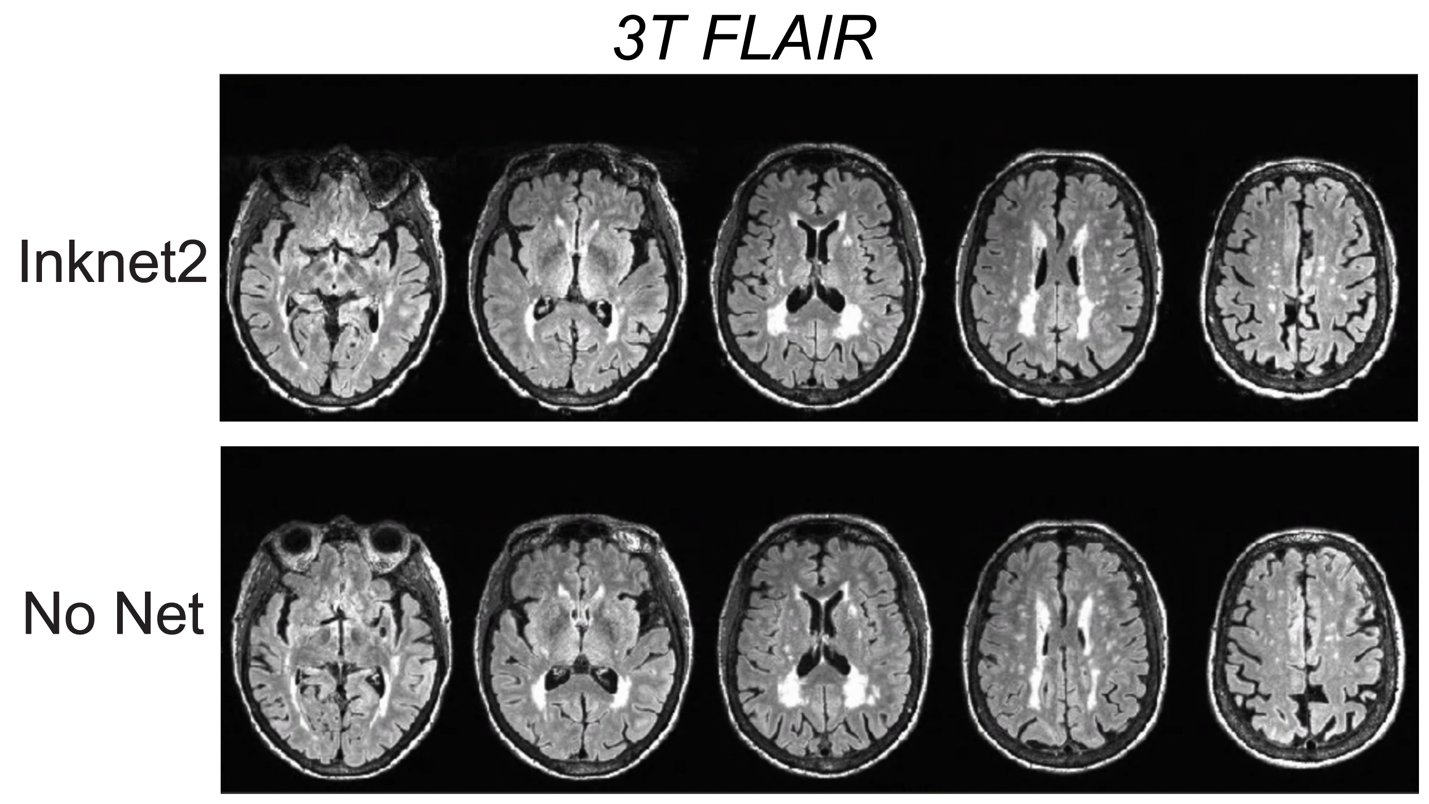
Supplemental Figure 6: Image quality and contrast are preserved in the 3T FLAIR with the Inknet2.** All images have identical color scales. Images were manually masked to remove the EEG electrodes outside the skull to maintain blinding to the condition.

**
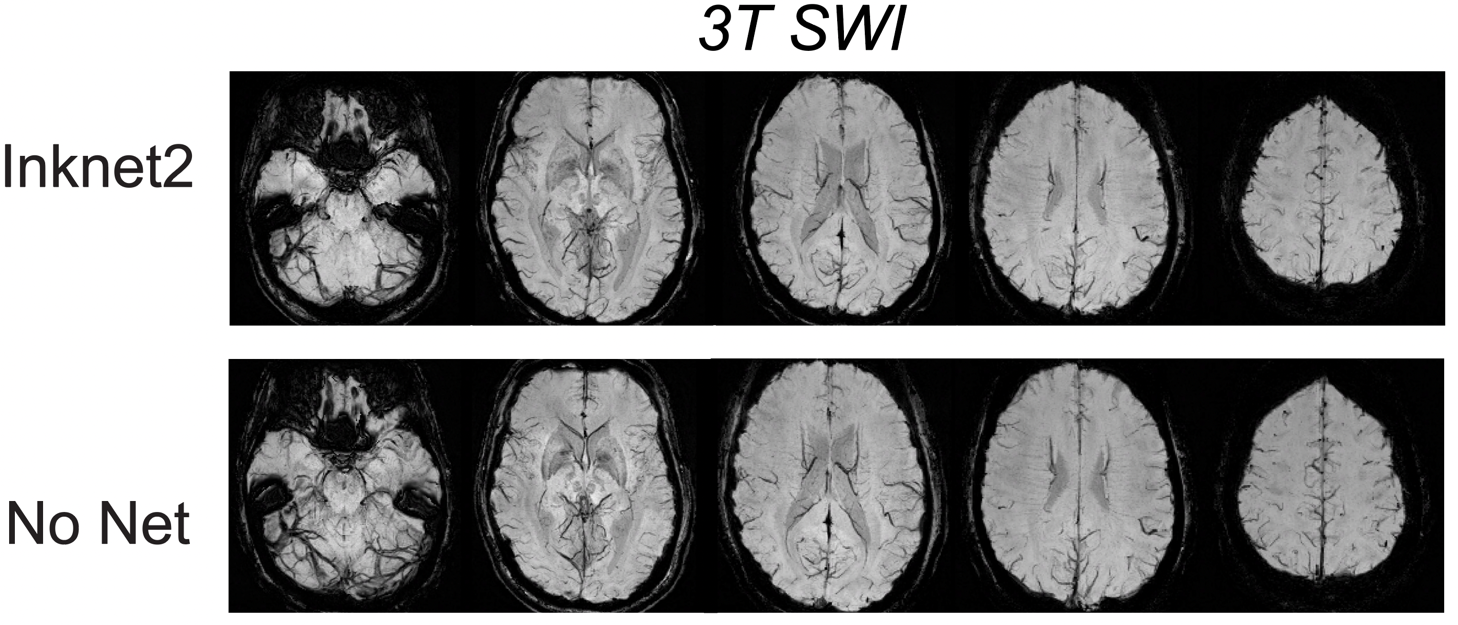
Supplemental Figure 7: Image quality and contrast are preserved in the 3T SWI with the Inknet2.** All images have identical color scales. Images were manually masked to remove the EEG electrodes outside the skull to maintain blinding to the condition.

**
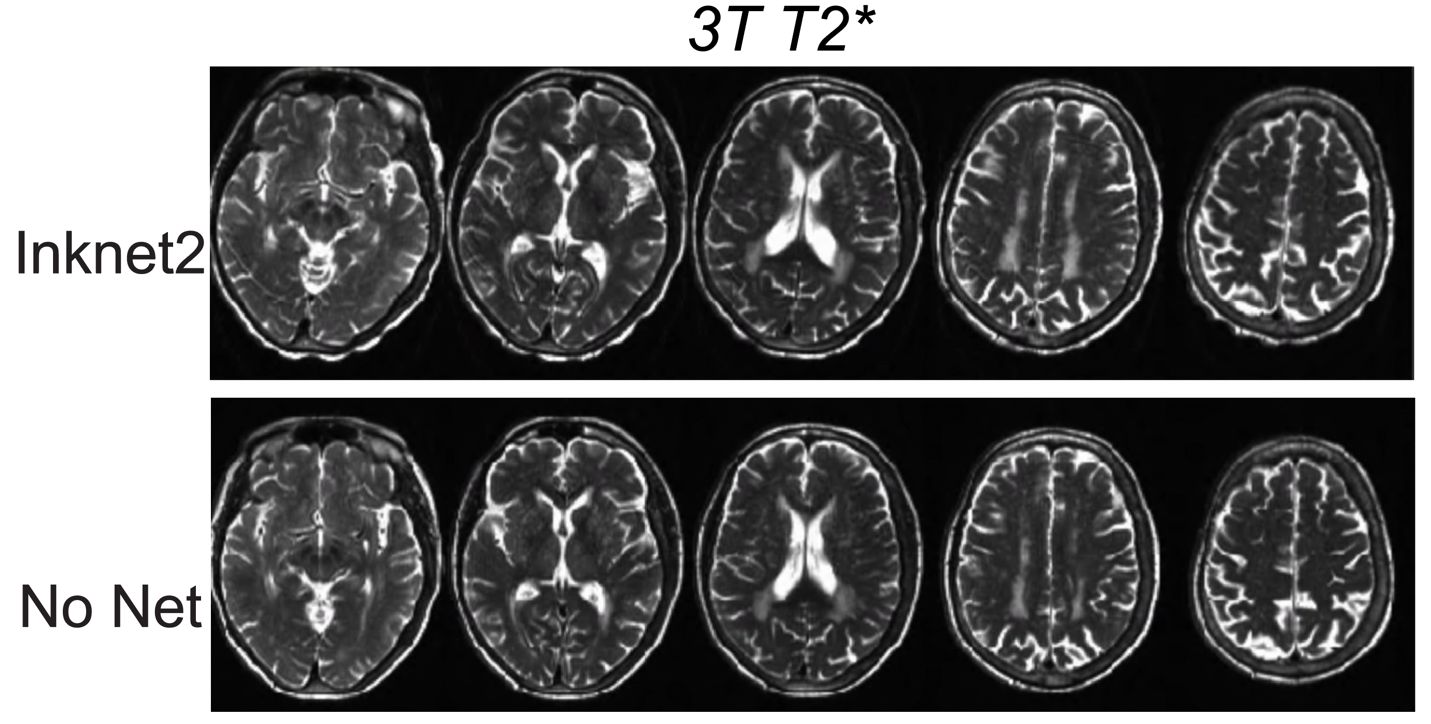
Supplemental Figure 8: Image quality and contrast are preserved in the 3T T2* with the Inknet2.** All images have identical color scales. Images were manually masked to remove the EEG electrodes outside the skull to maintain blinding to the condition.

**
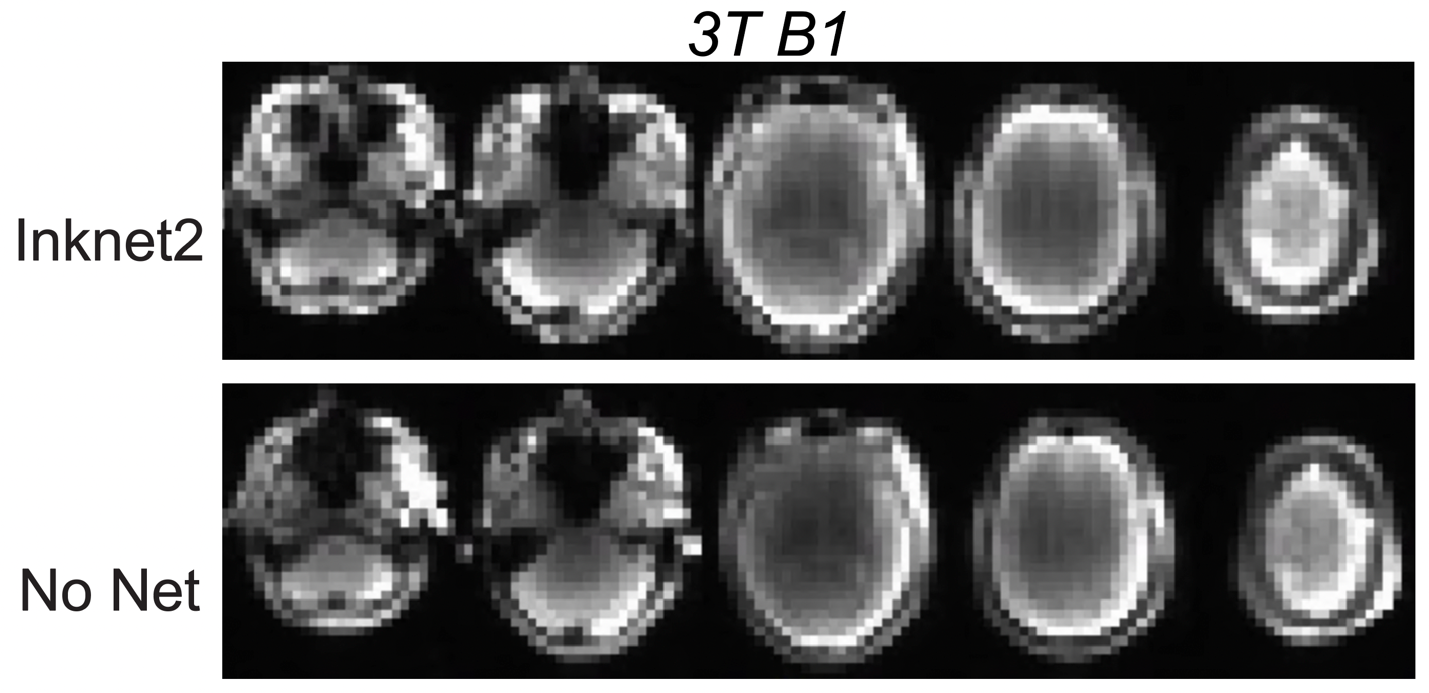
Supplemental Figure 9: Image quality and contrast are preserved in the 3T B1 with the Inknet2.** All images have identical color scales. Images were manually masked to remove the EEG electrodes outside the skull to maintain blinding to the condition.

**Supplemental Tables:**

**Supplemental Table 1: Likert Scale for neuroradiologist assessment**

| Likert Scale | | | | |
| --- | --- | --- | --- | --- |
| 1 | 2 | 3 | 4 | 5 |
| Extremely poor: *major artifacts exist and the images are not clinically useful* | **Poor:**  *major artifacts exist and clinical use is, therefore, not advised* | **Average**:  *average moderate artifacts detected with minor consequences for clinical useability* | **Good:**  *it contains minor artifacts that do not adversely affect clinical use* | **Excellent:**  *no artifacts* |
